# Clinical and CT scan findings in patients with COVID-19 pneumonia: a comparison based on disease severity

**DOI:** 10.1186/s43168-022-00142-w

**Published:** 2022-06-25

**Authors:** Sonia Hesam-Shariati, Susan Mohammadi, Morteza Abouzaripour, Behzad Mohsenpour, Bushra Zareie, Hana Sheikholeslomzadeh, Fahimeh Rajabi, Mohammad Bakhtiar Hesam Shariati

**Affiliations:** 1 Faculty of Medicine, School of Medical Sciences, University of New South Wales, Sydney, Australia; 2 Department of Radiology, Faculty of Medicine, Kurdistan University of Medical Sciences, Sanandaj, Iran; 3 Department of Anatomical Sciences, Faculty of Medicine, Kurdistan University of Medical Sciences, Sanandaj, Iran; 4 Department of Infectious Disease, Faculty of Medicine, Kurdistan University of Medical Sciences, Sanandaj, Iran; 5 Department of Epidemiology, School of Public Health, Hamadan University of Medical Sciences, Hamadan, Iran; 6 Department of Nursing Management, Tohid Hospital, Kurdistan University of Medical Sciences, Sanandaj, Iran; 7 Department of Radiology, Besat Hospital, Kurdistan University of Medical Sciences, Sanandaj, Iran

**Keywords:** COVID-19, Computed tomography, SARS-CoV-2, Acute respiratory disease, Pneumonia, Clinical features

## Abstract

**Background:**

The SARS-CoV-2 can cause severe pneumonia and highly impact general health. We aimed to investigate different clinical features and CT scan findings of patients with COVID-19 based on disease severity to have a better understanding of this disease.

**Methods:**

Ninety patients with coronavirus were divided into three categories based on the severity of the disease: mild/moderate, severe, and very severe. Clinical, laboratory, and CT scan findings of the patients were examined retrospectively. Any association between these features and disease severity was assessed.

**Results:**

The mean age and duration of hospitalization of patients increased with increasing the severity of the disease. The most common clinical symptoms were shortness of breath, cough, and fever. As the severity of the disease increased from mild/moderate to very severe, there was an increase in neutrophil counts and a decrease in lymphocytes and white blood cells (WBC) showing excessive inflammation associated with severe forms of COVID-19. Subpleural changes (81%) and ground-glass opacification/opacity (GGO) lesions (73%) of the lung were the most common features in CT images of COVID-19 patients, and interlobular septal thickening (10%) was the lowest CT feature among patients. Regarding the affected parts of the lung in COVID-19 patients, bilateral, peripheral, and multiple lesions had the highest prevalence.

**Conclusions:**

It has been shown that clinical, laboratory, and CT scan findings varied in COVID-19 patients based on disease severity, which need to be considered carefully in timely diagnosis and treatment of this illness.

## Introduction

In December 2019, the new coronavirus, which later named acute respiratory syndrome coronavirus 2 (SARS-CoV-2), was detected in Wuhan, China. SARS-CoV-2 leads to respiratory problems, which on February 11, 2020 was officially named coronavirus disease 2019 (COVID-19) by the World Health Organization (WHO) [1–3]. Numerous studies have shown that SARS-CoV-2 can contaminate human respiratory epithelial cells via an interplay between viral S protein and angiotensin-converting enzyme 2 (ACE2) on human cells; therefore, SARS-CoV-2 has a high potential to infect humans [4, 5].

The diagnosis of coronavirus disease is mainly dependent on the results of viral nucleic acid, which has high specificity but low sensitivity. According to a recent study, 50% of patients diagnosed with COVID-19 did not have any fever in the early stages of the disease [6], and even in the first few times of nucleic acid tests, negative results have been reported [5, 7]. Numerous studies have shown that simultaneous use of radiological findings (CT scans) and clinical findings can help in the early detection of COVID-19 [5, 8]. Many efforts have been made to collect documents in this area which showed CT scan of the chest as one of the most important methods in diagnosing COVID-19 pneumonia. Various forms of coronavirus-induced destruction of the lung parenchyma have been reported in radiological images, including fibrous stripes, interlobular septal thickening, irregular solid nodules, and ground-glass opacities [9–11].

However, previous studies have not specifically analyzed both clinical and CT scan findings based on disease severity. To further improve the understanding and early diagnosis of COVID-19, we retrospectively examined 90 patients admitted to hospitals in Sanandaj, Kurdistan Province in Iran, and classified them into three groups based on the severity of the disease. Clinical symptoms and features of pulmonary CT imaging were analyzed in each group.

## Materials and methods

### Study participants

In this study, 90 patients were diagnosed with COVID-19 in two hospitals in Sanandaj, Iran. Inclusion criteria for the patients were as follows: (1) positive epidemiological history of contact with definite or probable case, (2) detection of SARS-CoV-2 in nucleic acid reverse transcriptase reverse polymerase chain reaction of nose and throat swab, and (3) have had at least one CT scan of the lung.

COVID-19 patients were categorized in three different groups based on the severity of their illness: mild/moderate, severe, and very severe. These categories were based on guidelines for diagnosis and treatment of COVID-19 made by the Ministry of Health and Medical Education of Iran. Multiple factors including oxygen saturation, radiological findings, and clinical symptoms were assessed by clinicians to classify the patients. According to this classification, patients were either outpatient, hospitalized, or admitted to intensive care units (ICU) [12, 13].

### Data collection

Clinical parameters such as age, sex, hospitalization days, underlying diseases and conditions (hypertension, diabetes, heart disease, hypothyroidism, chronic obstructive pulmonary disease (COPD), kidney disease, malignancy, epilepsy and any history of surgery), clinical symptoms, laboratory findings, and chest CT scan were collected and evaluated. Data were collected between May 10 and November 5, 2020. Epidemiological, clinical, laboratory, and radiological data were obtained through patients’ medical records. We obtained any unavailable data through direct contact with physicians and other healthcare providers.

### Real-time polymerase chain reaction

According to the protocols for monitoring pneumonia caused by COVID-19 infection and instructions for laboratory diagnosis of coronavirus [14, 15], SARS coronavirus (SARS-CoV) was detected using commercial kits or COVID-19 rRT-PCR kit.

### Image acquisition

All CT scan images of patients were obtained in supine position using a Siemens CT scan machine. The parameters used for these images were as follows: detector collimation widths 64 × 0.6 mm, 128 × 0.6 mm, 64 × 0.6 mm, and 64 × 0.6 mm and tube voltage 120 kV. The images were reconstructed with a cutoff thickness of 1 mm. The reconstructed images were transferred to the hospital’s image archiving and communication systems (PACS) after processing.

### Image evaluation

The reconstructed CT images were transferred to the hospital archive and communication system (PACS) and were examined by a radiologist. Features of CT imaging were focused on the following aspects:

(A) Distribution of the lesion: left, right, or bilateral lungs
(B) Lesion site: peripheral, central, or both
(C) Lesion characteristics: crazy paving pattern, linear opacities, interlobular septal thickening, vascular enlargement, consolidation, rounded opacities, diffuse confluent, and patchy, ground-glass opacity, organizing pneumonia, subpleural curvilinear line, reticular pattern, pleural changes, pleural effusion, and multiple patchy areas of ground-glass opacities and peribronchovascular.

### Statistical analysis

Any association between the severity of the disease (mild/moderate, severe, and very severe) and clinical and demographic characteristics was examined. In addition, association between CT scan findings and illness severity as well as the outcome of the disease (death or recovery/discharge) was investigated. For the latter analysis, five patients were excluded since they were hospitalized at the time of our study. Chi-square tests for qualitative variables and one-way analysis of variance (ANOVA) were used to assess the relationship between disease severity and demographic, clinical, and laboratory findings. All the analysis was conducted in STATA 14 software, and a *p*-value less than 0.05 was considered as significant.

### Ethics

This research has been approved by the Research Center of Kurdistan University of Medical Sciences with the file number IR.MUK.REC.1399.019.

## Results

### Demographic characteristics of cases

Ninety patients with coronavirus were examined in this study. The frequency distribution was the same for both sexes. Mean (standard deviation) age of the patients was 60.41 (16.55); (men = 58.14 (18.14); women = 62.74 (14.60)) years. The frequency distribution of patients based on the severity of the disease in the three categories of mild/moderate, severe, and very severe was 40 (49.38%), 30 (37.04%), and 11 (13.58%), respectively. The mean age of patients slightly increased with increasing the severity of the disease from mild/moderate to very severe (Table 1). Overall, 75 (83%), 5 (6%), and 10 (11%) of the patients were treated in an outpatient setting, hospitalized, and died, respectively. The mean (standard deviation) of hospitalization days was 6.80 (5.33) which was significantly increased (*p*-value = 0.013) with increasing the severity of the disease (Table 1).

**Table 1:** Illness severity based on clinical and laboratory findings and demographic characteristics

	Illness severity			*p*-value†
	**Mild/moderate**	**Severe**	**Very severe**	
Sex; frequency (%)				
Male	17 (41.46)	16 (39.02)	8 (19.51)	0.193
Female	23 (57.50)	14 (35.00)	3 (7.50)	
Age; mean (SD)	57.97 (17.14)	62.10 (15.95)	65.63 (17.18)	0.859
Hospitalization days; mean (SD)	4.45 (3.30)	9.83 (5.65)	10.60 (7.49)	0.013
Initial symptoms: frequency (%)				
Fever	29 (59.18)	15 (30.61)	5 (10.20)	0.089
Cough	35 (49.30)	26 (36.62)	10 (14.08)	0.934
Sputum	6 (66.67)	2 (22.22)	1 (11.11)	0.533
Muscular pain	17 (47.22)	13 (36.11)	6 (16.67)	0.767
Tachypnea	36 (48.00)	28 (37.33)	11 (14.67)	0.523
Laboratory test; mean (SD)				
WBC (*10^3^)	7.36 (10.46)	11.23 (16.80)	12.55 (15.65)	0.075
Creatinine	1.13 (0.59)	1.17 (0.53)	1.24 (0.87)	0.300
AST (U/L)	20.30 (4.97)	33.77 (24.09)	25 (3.61)	0.698
Platelet	212 (101.49)	213.04 (101.51)	153.27 (72.37)	0.694
Lymphocyte	0.28 (0.11)	0.35 (0.30)	0.24 (0.29)	0.008
Neutrophil count *10^3^ L	0.65 (0.13)	0.76 (0.19)	0.84 (0.08)	0.035
CRP (mg/L)	22.73 (16.65)	44.09 (29.90)	34.67 (30.14)	0.823
Glucose	145.45 (49.15)	137.67 (64.45)	222.63 (108.93)	0.498
PTT	29.28 (3.56)	30.34 (6.27)	32.78 (11.03)	0.332
Prothrombin time	13.28 (3.76)	13.51 (3.04)	13.26 (1.32)	0.490
LDH (U/L)	608.38 (724.34)	714.62 (346.81)	803.00 (397.04)	0.910
Comorbidities; frequency (%)				
Diabetics	5 (35.71)	6 (42.86)	3 (21.43)	0.458
Hypertension	11 (47.83)	9 (39.13)	3 (13.04)	0.970
Cardiovascular disease	6 (46.15)	5 (38.46)	2 (15.38)	0.962
COPD	3 (60.00)	1 (20.00)	1 (20.00)	0.704
Hypothyroidism	1 (33.33)	3 (66.66)	0 (0.00)	0.516

^†^*p*-value from Pearson’s chi-squared test statistics and one-way ANOVA between illness severity and clinical and demographic characteristics. *WBC* white blood cell counts, *AST* aspartate aminotransferase, *CRP* C-reactive protein, *LDH* lactate dehydrogenase, *COPD* chronic obstructive pulmonary disease

### Disease characteristics of patients

The most common symptoms of the coronavirus disease were shortness of breath (*n* = 83; 92.22%), cough (*n* = 78; 86.67%), and fever (*n* = 54; 60%), respectively. A total of 17% and 15% of people with symptoms of muscle pain and shortness of breath experienced more severe disease, while the severity of the disease in about 67% and 60% of patients with sputum and fever was mild to moderate. Regarding the underlying diseases, more than 64% of patients with diabetes experienced COVID-19 severely (severe or very severe). This amount of severity of COVID-19 was experienced in 52% of patients with hypertension. The prevalence of patients with COPD or hypothyroidism among patients with coronavirus was 5% and 4%, respectively (Table 1). In addition, the prevalence of AIDS, acute kidney disease, history of surgery, malignancy, and epilepsy in COVID-19 patients was examined, which were all less than 4%.

### Features on computed tomography images

Examining CT scan of patients showed subpleural sparing with 81.11% and ground-glass opacification/opacity (GGO) with 73.33% as the most common CT manifestation and interlobular septal thickening with 10% as the lowest characteristic among patients with COVID-19 (Table 2). A total of 33%, 18%, and 17% of patients with interlobular septal thickening, multiple patchy areas of ground-glass opacities, and diffuse, respectively, had the highest severity of the disease. In addition, 50% of the individuals with interlobular septal thickening died, and there was a significant difference between the frequency of these characteristics in deceased and recovered patients (*p*-value < 0.001). In addition, more than 20% of patients with peribronchovascular and those with signs of viral pneumonia on CT scans have died. On the other hand, all the patients with organizing pneumonia and vascular enlargement recovered. The prevalence of recovery for patients with all the other features on CT scan (Table 2) was more than 80%.

**Table 2:** Illness severity and outcome of the disease based on CT scan characteristics

**CT characteristics**	**Illness severity**				***p*-value†**	**Outcome of the disease**		***p*-value‡**
	**Frequency (%)**	**Mild/moderate**	**Severe**	**Very severe**		**Alive**	**Death**	
Crazy paving pattern	23 (25.66)	7 (35.00)	10 (50.00)	3 (15.00)	**0.308**	20 (90.91)	2 (9.09)	**0.651**
Interlobular septal thickening	9 (10.00)	2 (33.33)	2 (33.33)	2 (33.33)	**0.329**	4 (50.00)	4 (50.00)	** < 0.001**
Vascular enlargement	18 (20.00)	11 (64.71)	4 (23.53)	2 (11.76)	**0.344**	16 (100.00)	0 (0.00)	**0.105**
Consolidation	33 (36.67)	16 (50.00)	11 (34.38)	5 (15.63)	**0.875**	26 (86.67)	4 (13.33)	**0.740**
Rounded opacities	27 (30.00)	9 (36.00)	12 (48.00)	4 (16.00)	**0.267**	24 (88.89)	3 (11.11)	**0.898**
Diffuse confluent and patchy	37 (41.11)	17 (50.00)	13 (38.24)	4 (11.76)	**0.919**	31 (88.57)	4 (11.43)	**0.936**
Air bronchogram	26 (28.89)	14 (53.85)	8 (30.77)	4 (15.38)	**0.722**	20 (83.33)	4 (16.67)	**0.379**
Ground-glass opacity	66 (73.33)	31 (51.67)	22 (36.67)	7 (11.67)	**0.645**	55 (87.30)	8 (12.70)	**0.651**
Organizing pneumonia	14 (15.56)	7 (53.85)	6 (46.15)	0 (0.00)	**0.285**	14 (100.00)	0 (0.00)	
Subpleural curvilinear line	19 (21.11)	10 (58.82)	6 (35.29)	1 (5.88)	**0.510**	17 (94.44)	1 (5.56)	**0.357**
Viral pneumonia	11 (12.22)	8 (80.00)	1 (10.00)	1 (10.00)	**0.104**	8 (80.00)	2 (20.00)	**0.390**
Pleural changes	20 (22.22)	9 (52.94)	6 (35.29)	2 (11.76)	**0.939**	17 (94.44)	1 (5.56)	**0.357**
Pleural effusion	18 (20.00)	7 (50.00)	4 (42.86)	1 (7.14)	**0.714**	15 (93.75)	1 (6.25)	**0.447**
Multiple patchy areas of ground-glass opacities	49 (54.44)	23 (51.11)	14 (31.11)	8 (17.78)	**0.311**	39 (82.98)	8 (17.02)	**0.094**
Peribronchovascular	17 (18.89)	7 (50.00)	5 (35.71)	2 (14.29)	**0.992**	11 (78.57)	3 (21.43)	**0.219**
Diffuse	24 (26.67)	11 (47.83)	8 (34.78)	4 (17.39)	**0.817**	18 (81.82)	4 (18.18)	**0.278**
Subpleural	73 (81.11)	33 (49.25)	24 (35.82)	10 (14.93)	**0.714**	62 (88.57)	8 (11.43)	**0.835**

^†^*p*-value from Pearson’s chi-squared test statistics and one-way ANOVA between illness severity and CT characteristics

^‡^*p*-value from Pearson’s chi-squared test statistics between outcome of the disease and CT characteristics

The frequency distribution of affected parts of the lung in all the patients has been shown in Table 3. It has been revealed that more than 91% of all the patients (both recovered and dead) had peripheral and bilateral lesions, and 90% of them had multiple lesions in the lung CT scan. In addition, all the COVID-19 patients who died had bilateral, peripheral, and multiple lesions at the same time.

**Table 3:** Frequency distribution of affected parts of the lung in coronavirus patients and deceased patients

Lesion	COVID-19 group (*n* = 90) Frequency (%)	Death group (*n* = 10) Frequency (%)
Unilateral	2 (2.22)	0 (0.00)
Bilateral	82 (91.11)	10 (100.00)
Peripheral	83 (92.22)	10 (100.00)
Central	36 (40.00)	4 (40.00)
Single	1 (1.11)	0 (0.00)
Multiple	81 (90.00)	10 (100.00)
Diffuse	27 (30.00)	4 (40.00)

### Laboratory abnormalities in COVID-19 patients

As the severity of the disease increased from mild/moderate to very severe, there was a statistically significantly decrease in lymphocyte counts (*p*-value = 0.008) and an increase in neutrophil counts (*p*-value = 0.035). White blood cells (WBC) also showed an increasing trend (*p*-value = 0.075) with increasing the severity of disease. Creatinine, aspartate aminotransferase (AST), C-reactive protein (CRP), mean glucose level, partial thromboplastin time (PTT), and lactate dehydrogenase (LDH) increased with increasing the severity of the disease, while platelets decreased from 212 to 153, although none of these features was statistically significant (Table 1).

## Discussion

COVID-19 pneumonia, started in Wuhan, China, declared a public health emergency by the World Health Organization (WHO) on January 30, 2020 [16]. The cause of the infection was a new coronavirus (SARS-CoV-2) that has been reported to cause symptomatic and asymptomatic infections. So far, studies have shown that the main routes of transmission of the virus are respiratory droplets and direct contact. The incubation period of this viral disease is generally reported to be 3–7 days but can take up to 14 days [17, 18].

In this study, we examined clinical, laboratory, and CT scan findings of 90 patients with COVID-19 retrospectively. It has been shown that clinical and CT scan findings of patients were different across different ages, and there was a slightly higher mean age in patients with a more severe disease. There was also a direct relationship between the average duration of hospitalization and the severity of the disease. Clinical manifestations can indicate patient’s physical condition, and the findings on CT images often indicate clinical severity. Studying clinical features and CT scan images can be useful in understanding the differences in disease features between different age groups which can be useful in clinical diagnosis and treatment decisions. Studies have shown that elderly patients with underlying diseases are more likely to have impaired physical activity and weakened immune systems and therefore more susceptible to the effects of coronavirus [19].

According to studies, the most common symptoms of COVID-19 are fever, cough, and shortness of breath [20]. We also found shortness of breath (92.22%), cough (86.67%), and fever (60%) as the most common symptoms in patients. In addition, it has been shown that almost 17% and 15% of people with symptoms of muscle pain and shortness of breath have experienced more severe disease, while the severity of the disease in people with symptoms of sputum and fever was milder than other people.

Several underlying diseases were examined in this study. It has been shown that most of the patients with diabetes and hypertension experienced severe or very severe forms of COVID-19. The severity of coronavirus disease based on the type of underlying disease has not been reported previously, but a number of studies have shown that the most common diseases associated with coronavirus are hypertension, diabetes, and coronary heart disease [21, 22]. Fang and his colleagues also showed that the severity of coronavirus disease was higher in people with underlying hypertension, diabetes mellitus, coronary heart disease, and cerebrovascular disease [23]. In addition to underlying diseases mentioned in Table 1, AIDS, acute kidney disease, surgical history, malignant tumors, and epilepsy were examined in this study. However, their prevalence was less than 4%.

In our study, with increasing the severity of COVID-19 from mild/moderate to very severe, WBC and neutrophils increased. In a study conducted by Pozdnyakova and his colleagues on 90 patients with COVID-19, it has been concluded that all the patients had significant numerical and morphological changes in WBC, and there was also a difference between mild and severe disease. Based on their results, more severe disease was associated with significant increase in neutrophils and lymphopenia, which intensified in very severe patients. The abnormal WBC morphology, which is more prominent in monocytes and lymphocytes, was associated with milder disease, and the changes disappeared as the disease progressed [24]. We also observed an indirect relationship between lymphocytes counts and the severity of COVID-19. Our findings confirmed previous studies which reported an increase in neutrophils and a decrease in lymphocytes in severe or non-survival COVID-19 patients which might be due to severe inflammation caused by COVID-19 [25, 26].

In terms of CT characteristics, subpleural changes had the highest frequency (81%) in patients with COVID-19 (Table 2). GGO with 73% was the second noticeable imaging finding (Table 2), indicating that coronavirus pneumonia is mainly based on interstitial lung secretion [10, 27]. This means that the pathological mechanism of COVID-19 is through dilatation and occlusion of alveolar septal capillaries, fluid secretion in the alveolar cavity, and interstitial edema of the leaflet blade [28]. There was not a significant association between subpleural lesions or GGO and the severity or outcome of the disease in our study (Table 2). Studies have shown that patients with the highest severity of COVID-19 have features such as thickening of the interlobular septum, several areas of ground-glass opacity, and diffusion on CT scan images. In addition, the frequency distribution of affected parts of the lung in these patients is more in the form of peripheral lesions [29]. In this study, it was also found that most of the patients with characteristics such as interlobular septal thickening, multiple patchy areas of ground-glass opacities, and diffuse in CT scan images had severe or very severe forms of the disease (Table 2). It seems that coronavirus tend to colonize in bilateral and peripheral areas and in the form of multiple lesions (Table 3). In all deceased individuals, bilateral, peripheral, and multiple lesions were observed. Previous studies also revealed bilateral and peripheral involvement and GGO as the most common CT abnormalities in COVID-19 patients [30]. There is a strong correlation between different age groups, disease severity, and number of affected lobes [29]. We also observed that the average age of patients slightly increases with increasing the severity of the disease. The lungs of elderly patients are more involved in interstitial changes, which may indicate that the lungs of the elderly are more affected by viral infections and the viruses spread easily in them [31, 32].

In conclusion, it has been shown that clinical, laboratory, and CT scan findings in COVID-19 are different based on the severity of disease. A higher count of neutrophils compared to lymphocytes was revealed in more severe forms of the disease showing higher inflammation rate. GGO and subpleural were the most common CT scan features. Lesions were multiple and mostly affected bilateral and pepripheral parts. Underlying diseases also impact the severity of COVID-19 and should be considered. Overall, a better understanding of these differences can be valuable in the timely and effective diagnosis and treatment of patients with coronavirus. Most importantly in elderly, whose conditions are likely to be more severe, CT scan and clinical features can provide important information to speed up the diagnosis and treatment.

## Data Availability

I have presented the data of the patients in the manuscript as tables.
